# Hemofiltration compared to hemodialysis for acute kidney injury: systematic review and meta-analysis

**DOI:** 10.1186/cc11458

**Published:** 2012-08-06

**Authors:** Jan O Friedrich, Ron Wald, Sean M Bagshaw, Karen EA Burns, Neill KJ Adhikari

**Affiliations:** 1 Department of Medicine, University of Toronto, Toronto, ON, M5G 2C4,Canada; 2 Department of Medicine, St. Michael's Hospital, 30 Bond Street, Toronto, ON M5B 1W8, Canada; 3 Critical Care Department, St. Michael's Hospital, 30 Bond Street, Toronto, ON M5B 1W8, Canada; 4 The Keenan Research Centre in the Li Ka Shing Knowledge Institute, St. Michael's Hospital, Toronto, ON M5B 1W8, Canada; 5 Interdepartmental Division of Critical Care, University of Toronto, Toronto, ON, M5B 1W8, Canada; 6 Division of Critical Care Medicine, Faculty of Medicine and Dentistry, University of Alberta, Edmonton, AB T6G 2B7, Canada; 7 Department of Critical Care Medicine and Sunnybrook Research Institute, Sunnybrook Health Sciences Centre, 2075 Bayview Avenue, Toronto, ON M4N 3M5, Canada

## Abstract

**Introduction:**

The objective of this systematic review and meta-analysis was to determine the effect of renal replacement therapy (RRT), delivered as hemofiltration vs. hemodialysis, on clinical outcomes in patients with acute kidney injury (AKI).

**Methods:**

MEDLINE, EMBASE and CENTRAL databases and conference abstracts were searched to June 2012 for parallel-group or crossover randomized and quasi-randomized controlled trials (RCTs) evaluating hemofiltration vs. hemodialysis in patients with AKI. Two authors independently selected studies and abstracted data on study quality and outcomes. Additional information was obtained from trial authors. We pooled data using random-effects models.

**Results:**

Of 6,657 citations, 19 RCTs (10 parallel-group and 9 crossover) met inclusion criteria. Sixteen trials used continuous RRT. Study quality was variable. The primary analysis included three parallel-group trials comparing similar doses of hemofiltration and hemodialysis; sensitivity analyses included trials comparing combined hemofiltration-hemodialysis or dissimilar doses. We found no effect of hemofiltration on mortality (risk ratio (RR) 0.96, 95% confidence interval (CI) 0.73 to 1.25, *P *= 0.76; three trials, n = 121 (primary analysis); RR 1.10, 95% CI 0.88 to 1.38, *P *= 0.38; eight trials, n = 540 (sensitivity analysis)) or other clinical outcomes (RRT dependence in survivors, vasopressor use, organ dysfunction) compared to hemodialysis. Hemofiltration appeared to shorten time to filter failure (mean difference (MD) -7 hours, 95% CI (-19,+5), *P *= 0.24; two trials, n = 50 (primary analysis); MD -5 hours, 95% CI (-10, -1), *P *= 0.01; three trials, n = 113 (including combined hemofiltration-hemodialysis trials comparing similar doses); MD -6 hours, 95% CI (-10, -1), *P *= 0.02; five trials, n = 383 (sensitivity analysis)). Data primarily from crossover RCTs suggested that hemofiltration increased clearance of medium to larger molecules, including inflammatory cytokines, compared to hemodialysis, although almost no studies measured changes in serum concentrations. Meta-analyses were based on very limited data.

**Conclusions:**

Data from small RCTs do not suggest beneficial clinical outcomes from hemofiltration, but confidence intervals were wide. Hemofiltration may increase clearance of medium to larger molecules. Larger trials are required to evaluate effects on clinical outcomes.

## Introduction

Severe acute kidney injury (AKI) occurs in approximately 6% of patients admitted to an intensive care unit (ICU) [1] and in up to 19% of patients with vasopressor-dependent septic shock [2]. For such individuals, mortality is approximately 60% [1], and survivors are at increased risk of requiring permanent renal replacement therapy (RRT) [3]. Two multicenter randomized controlled trials (RCTs) [4,5] and two meta-analyses [6,7] have demonstrated that increasing the dose of RRT above 20 to 25 mL/kg/h of effluent flow for continuous renal replacement therapy (CRRT) or increasing intermittent dialysis frequency beyond alternate days does not improve survival.

In addition to dose, the mode of clearance is also a modifiable component of the RRT prescription that may affect patient outcomes. Convective clearance and diffusive clearance, delivered by hemofiltration and hemodialysis, respectively, can be provided by all continuous and some intermittent RRT machines. Despite similar clearance of small molecules, hemofiltration is reported to achieve higher clearance of medium-sized to larger molecules compared to hemodialysis [8]. Consequently, it is postulated that hemofiltration might benefit critically ill patients with AKI by better clearing large toxic inflammatory cytokines [9]. In the absence of a large, suitably-powered, randomized trial demonstrating the superiority of one mode over the other, practice surveys have shown variability in mode selection among countries and regions [10-15]. Therefore, our objective was to conduct a systematic review and meta-analysis of all RCTs comparing the effects of convective clearance (using hemofiltration) to diffusive clearance (using hemodialysis) in patients with AKI on clinically important outcomes.

## Materials and methods

### Literature search

We searched OVID versions of MEDLINE, EMBASE Classic and EMBASE and the Cochrane Central Register of Controlled Trials (from inception to June 2012) without language restrictions using a previously described search strategy [6]. We also searched abstracts from critical care and nephrology professional society conferences, including: Society of Critical Care Medicine (2004 to 2012), European Society of Intensive Care Medicine (2001 to 2011), International Symposium of Intensive Care and Emergency Medicine (2004 to 2012), American Thoracic Society (2004 to 2012), American College of Chest Physicians (2003 to 2011), American Society of Nephrology (2003 to 2011), and the European Renal Association - European Dialysis and Transplant Association (2002 to 2012). We also searched bibliographies of included studies and personal files. Two reviewers independently reviewed all citations and retrieved the full text of any citation considered potentially relevant by either reviewer. We attempted to contact selected authors of included studies for clarification of methods and to obtain additional data, where required.

### Study selection

Two unblinded reviewers assessed full-text reports and included studies meeting the following criteria: (1) design: either parallel-group (patients assigned to only one treatment) or cross-over (each patient received both treatments in random order) randomized or quasi-randomized (for example, assigning patients in alternating fashion or by hospital registry number) controlled trial, (2) population: adult or post-neonatal pediatric patients with AKI requiring RRT, (3) intervention: hemofiltration compared to hemodialysis, with both modes applied using continuous or intermittent RRT; trials comparing continuous to intermittent RRT were excluded, (4) outcomes: all-cause mortality (primary outcome) or other clinically important outcomes (see below) for the parallel-group trials; or group-specific filter duration, or clearance or plasma concentration measurements of cleared substances for both parallel-group and crossover trials. We also included trials of hemodiafiltration (that is, combined dialysis-filtration) compared to either hemodialysis or hemofiltration in sensitivity analyses as explained below.

### Data abstraction and validity assessment

Two unblinded reviewers independently abstracted data from included trials, including study population (number of centers, age, gender, illness acuity proportion with oliguria and sepsis, baseline creatinine and urea), RRT methods (modality, dose, duration of and criteria for starting/stopping study RRT), outcomes (mortality, RRT duration, RRT dependence in survivors, hemodynamics (for example, vasopressor doses), evolution of organ dysfunction, filter duration, measured clearances and plasma concentrations of metabolites) and study quality (including method of sequence generation and allocation concealment, intention-to-treat analysis, loss to follow-up for the outcome of mortality, and early trial stopping for efficacy before the planned enrollment was completed). For the crossover trials we also assessed whether carry-over effects and washout periods were addressed and whether paired data analyses were performed [16]. Disagreements between reviewers regarding study selection and data abstraction that remained after author contact were resolved by consensus.

### Data analysis

Our primary outcome was all-cause hospital mortality and, if not available, then mortality at 90, 60, 30 or 28 days after randomization, at ICU discharge or after stopping RRT (in descending order of preference). Secondary outcomes included RRT dependence among survivors at the latest time point available (with the same preferred order of time point as for the mortality analysis), RRT duration until renal recovery or death, filter duration, clearance of selected solutes and plasma concentration measurements.

Binary outcomes are reported as risk ratios (RR) and continuous outcomes using weighted mean differences (MD, a measure of absolute change) or ratio of means (RoM, a measure of relative change) [17]. We used Review Manager 5.1 (The Cochrane Collaboration, Oxford, England, UK) to calculate pooled outcome measures. We considered (two-sided) *P *<0.05 as statistically significant and reported individual trial and summary results with 95% confidence intervals (CIs). We used random-effects models, which incorporate between-trial heterogeneity and give wider confidence intervals when heterogeneity is present, to pool data. We assessed statistical heterogeneity among trials using *I^2^*, the percentage of total variability across studies due to heterogeneity rather than chance [18,19], and used published thresholds to ascribe low (*I^2 ^*= 25 to 49%), moderate (*I^2 ^*= 50 to 74%), and high (*I^2 ^*≥75%) heterogeneity [19]. Continuous variables are expressed as mean ± standard deviation, unless otherwise indicated. Because the crossover trials generally did not report within-patient differences, we used the unpaired group-specific means to pool data, recognizing that this approach reduces the statistical power to detect differences [20].

For each outcome, the primary analysis included only trials in which one group was treated only with hemofiltration and the other group only with hemodialysis, with both groups receiving RRT providing similar (that is, within 20%) small-molecule clearances. In assessing equivalency of doses, we determined whether trial authors adjusted for the reduced dose of hemofiltration that results from blood dilution by pre-filter replacement fluid, where applicable. In sensitivity analyses for each outcome, we included data from trials in which doses differed by greater than 20% between the convection and diffusion groups, and from trials comparing combined filtration-dialysis modes to either filtration or dialysis. For trials in which one group was treated with combined filtration-dialysis, this group was considered either as filtration (if compared to a dialysis-only group) or dialysis (if compared to a filtration-only group).

To assess for publication bias we planned to visually examine a funnel plot of study precision versus treatment effect on mortality for evidence of asymmetry, assuming ≥5 trials in the analysis.

Because this study reports an analysis of published data, ethical approval was not required.

## Results

### Study flow

**Figure 1:**
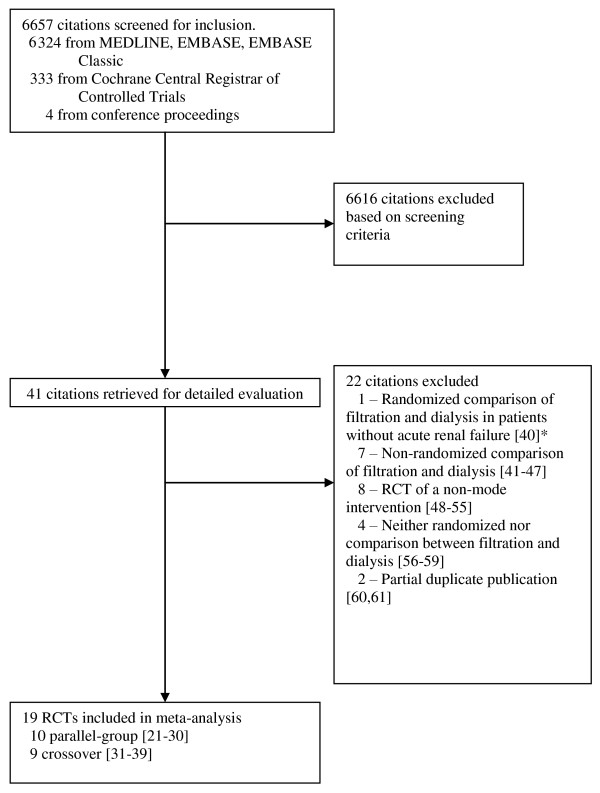
**Flow chart of study selection**. *In addition, author contact confirmed that mortality and other clinical outcomes not collected, and clearance rates not measured for this trial. Abbreviation: RCT, randomized controlled trial.

Our search strategy identified 6,657 citations, 6,324 from Medline, EMBASE Classic and EMBASE and 333 from the Cochrane Central Register of Controlled Trials. We retrieved 41 articles for detailed evaluation, of which 19 studies met criteria for inclusion [21-39] and 22 were excluded [40-61] (Figure 1). They included 10 parallel-group trials [21-30] and 9 crossover trials [31-39]. Among the parallel-group trials, authors of 9 included trials [21,23-30] provided additional methodological or clinical data, and the author of the 10^th ^trial [22] informed us that no additional information was available. In addition, authors of two studies informed us either that the vast majority of enrolled randomized patients did not have renal failure [40], or that it was not a randomized controlled trial [41] (reference [62] is the full paper for the abstract reference [41]), leading to exclusion of these studies.

### Description of included studies (Table 1)

Enrolled patients had high illness severity. The mean or median acute physiology and chronic health evaluation (APACHE) II score [63] was ≥20 or simplified acute physiology score (SAPS) II [64] was ≥60, as reported in 8 of 10 parallel-group trials [21-27,29] and 3 of 9 crossover trials [37-39]. One parallel-group trial reported only the mean sequential organ failure assessment (SOFA) [65] score of 16 [30]. Where reported, the vast majority of patients required mechanical ventilation or vasopressors [21,23,25,29-32,35,36]. AKI was defined by abnormal biochemistry (serum creatinine or urea) or a complication of oliguria (such as volume overload, hyperkalemia, acidosis or uremic symptoms) in 7 of 10 parallel-group trials [21,23-26,29,30] and 3 of 9 crossover trials [34,35,38]. The remaining nine trials enrolled patients with AKI requiring RRT without reporting specific AKI criteria [22,27,28,31-33,36,37,39]. Five trials explicitly excluded patients with chronic kidney disease, defined by pre-morbid creatinine or creatinine clearance [23,25,26,29] or recent dialysis [30],

**Table 1:** Patient characteristics in included trials

						Illness Acuity									
											
Trial	Patients,n	Centers,n	Age,yr	Male%	Surg%	APACHE II	SAPS II	MODS or SOFA	Ventilated%	Vasopressors%	**Oliguria**, %	Cr,μM	Urea,mM	Sepsis,%	Mortality ^a^,%
***Parallel Group***															

Davenport 1993 [21] (medians)	20	1	33	45%		28			100%		100%^b^	646	18		84% ^h^

Alamartine 1994 [22]	13	1	n/r	n/r		22						566	30		n/a

Pettila 2001 [23]	39	1	48	82%		20		10 MODS	95%		61%^c^	446	31		42%

Morgera 2004 [24] (medians)	24	1	65	58%		31						254	44		50% (ICU)

Daud 2006 [25] (medians)	20	2	50	60%			66		100%			265	19	90%	85% (ICU)

Saudan 2006 [26]	206	1	63	61%		25		9 SOFA			37%^d^	428	30	60%	53% (90d)

Chang 2009^j ^[27]	96	1	65	57%		31		13 SOFA				238	19	100%	54% (28d)

Ratanarat 2009^k ^[28]	17	1	65												n/c

Ratanarat 2012 [29]	60	1	57	60%	0%	27			93%	67%		449	32	82%	47% (28d)

OMAKI 2012 [30]	77	6	61	61%				16 SOFA	95%	90%		261	22	82%	55%

***Crossover***															

Maher 1988 [31]	5	1	median 51	100%					80%	100%					

Alarabi 1992 [32]	13	1	60	62%	38%				92%	100%		453	36		

Jeffery 1994 [33]	10	1													

Kellum 1998 [34]	13 (10)^e^	1												100%	77(70) %^e^

Garcia-Fernandez 2000 [35]	40	1	60	65%	38%				58%	53%	70%^i^			48%	48%

Maxvold 2000 [36]	6	1	11	33%	17%					100%					50% ( undef)

Wynkel 2004 [37]	18	1	62		39%		60^g^								50% (30d)

Ricci 2006 [38] (medians)	15	1	50	67%	60%		61								

Davies 2008 [39]	45 (31)^f^	1	61 (57)	64% (77%)	38%	26									

Means, (medians where noted). ^a^Hospital mortality except where noted. Oliguria: ^b^<10 mL/h [21], ^c^<500 mL/24h [23], ^d^<200 mL/12 h [26], and ^i^<400 mL/24h [34]. ^e^For Kellum 1998 [34], 3/13 patients died prior to being crossed over to other arm. ^f^For Davies 2008 [39], 45 patients were randomized but only 31 patients crossed over and received both therapies. ^g^Wynkel 2004 [37] excluded SAPS II >= 85. ^h^Mortality obtained after author contact [21]. ^j^Chang 2009 [27] reported data from 65 patients in abstract form; data for 31 patients who were subsequently randomized provided after author contact. ^k^Author informed us that mortality was not recorded [28]. Abbreviations: APACHE II, acute physiology and chronic health evaluation II [63]; Cr, serum creatinine concentration in micromoles per litre; MODS, multiple organ dysfunction score [66]; n/a, not available after author contact; n/c, not collected; n/r, not reported; SAPS II, simplified acute physiology score II [64]; SOFA, sequential organ failure assessment score [65].

### Description of RRT interventions (Table 2)

Seven of the 10 parallel-group trials used continuous RRT [21,22,24-27,30-39], 1 trial used intermittent RRT [23] and 2 trials used sustained low efficiency RRT provided in sessions of eight hours each day [28,29]. Only three parallel-group trials, all using CRRT, (n = 24 [24], n = 20 [25], n = 78 [30]) compared exclusively hemofiltration to exclusively hemodialysis at similar small-molecule doses. Of these, one trial allocated patients to higher (2.5 L/h) or lower (1 L/h) dose continuous veno-venous hemofiltration (CVVH) or continuous veno-venous hemodialysis (CVVHD) in a 2 × 2 factorial design [24]. In six of the remaining seven parallel-group trials, patients treated with hemofiltration (n = 20 [21], n = 206 [26], n = 96 [27]) or hemodialysis (n = 13 [22], n = 39 [23], n = 60 [29]) were compared to a group treated with hemodiafiltration (hemofiltration and hemodialysis). Hemodiafiltration recipients received higher doses, except in three trials where doses in the two treatment arms were similar, although not corrected for lower clearance due to pre-filter addition of replacement fluid [23,27,29]. The seventh trial, which used sustained low efficiency RRT and was reported only in abstract form, randomized patients into three groups: two similar-dose groups of hemodialysis (n = 6) and hemodiafiltration (n = 5) and a lower-dose group of hemofiltration (n = 6) [28]. Only one parallel-group trial was multi-centered [30].

**Table 2:** Renal replacement intervention in the included trials

					Filtration only group		Combined filtration and dialysis group		Dialysis only group	
					
Trial	Cessation of study RRT	Filter	Anti-coagulation	Blood flow,mL/min	Mode^b^	Dose,^c ^L/h; Study RRT duration^j^	Mode^b^	Dose,^c ^L/h (or d); Study RRT duration^j^	Mode	Dose,^c ^L/h (or d); Study RRT duration^j^
***Parallel Group***										

Davenport 1993 [21]	Clinician	FH77 (Gambro)	UFH	n/r	CAVH (post)	0.9 L/h (median)	CAVHDF (post)	1 (D) + 0.4 (F) L/h (median)		

Alamartine 1994 [22]	Clinician	0.90 m^2 ^F50 poly-sulfone (Fresenius-Smad)	UFH	150			CVVHDF (pre)	1(D) + 2(F) L/h	CVVHD	1 L/h

Pettila 2001 [23]	Clinician	Polyflux 17	LMWH	250			IHDF (pre)	40 (F) + 128 (D) L/d; 23.5 ± 18.8 d	IHD	164 (D) + 4(F) L/d; 17.5 ± 9.2 d

Morgera 2004 [24]^d^	Maximum of 3 days	Polyflux P2SH 1.1 m^2^, 10 nm pore, 50 μm membrane thickness; 60 kDa cutoff	UFH	n/r	CVVH (post)	1 or 2.5 L/h; 2.8 d			CVVHD	1 or 2.5 L/h; 2.8 d

Daud 2006 [25]	Clinician	AN69 0.6 m^2^, changed daily	UFH	≥120	CVVH (pre)	2 L/h; 2.0 (IQR 5.1) d			CVVHD	1.7 L/h; 1.9 (IQR 2.2) d

Saudan 2006 [26]	Protocol	AN69 0.9 m^2 ^(Gambro) changed daily × 2 d	UFH	100 to 125	CVVH (pre)	1.8 L/h (25 mL/kg/h)	CVVHDF (pre)	3.1 L/h (24 (F) + 18 (D) mL/kg/h)		

Chang 2009 [27]	Clinician	multiflow 100 (Gambro)	UFH or no anticoag if high risk bleed	100 to 150	CVVH (pre)	2.4^k ^L/h (40 mL/kg/h); 7.4 ± 8.0 d	CVVHDF (pre)	2.4^k ^L/h (20 [F] + 20[D] mL/kg/h); 8.5 ± 8.9 d		

Ratanarat 2009 [28]^l^	Maximum 3 days	F40s or F80s^l^	n/r	200 to 250 or 250 to 300^l^	Sustained low efficency filtration (pre)	39 ± 6^l ^L/d (1.25 × body weight (in kg) mL/min)	Sustained low efficiency diafiltration (pre)	48 (F) + 96 (D) L/d	sustained low efficiency dialysis	144 L/d

Ratanarat 2012 [29]	Clinician	HF80S (1.8 m^2^, Kuf 55 mL/h × mm Hg) (Fresenius)	UFH	250			Sustained low efficiency diafiltration (pre)	48 (F) + 96 (D) L/d	Sustained low efficiency dialysis	144 L/d

OMAKI 2012 [30]	U/O>500 mL/12h, K <5.5 mM and HCO_3 _>18 mM; or stepdown to IHD when SOFA CV score <2 for >24h;	AN69 ST100 (1 m^2^) or ST150 (1.5 m^2^) (Gambro)	UFH or citrate or no anticoag.	≥150	CVVH (equally distributed pre/post)	3.0.L/h (2.7 L/h post equiv.) (34 mL/kg/h); 5 (IQR 3 to 7) d			CVVHD	3.1 L/h (incl. 0.2 L/h F post) (35 mL/kg/h); 4.5 (IQR 3 to 10.25) d

***Crossover***										

Maher 1988 [31]^e^	n/r	FH55 HF or AN69S HD (Gambro)	n/r	n/r	CAVH (n/r)	1 L/h			CAVHD	1 L/h

Alarabi 1992 [32]^f^	24 h	Polysulphone (Amicon AMD30)	UFH	n/r	CAVH (post)	0.833L/h (median)	CAVHDF (post)	0.9 D + 0.313 F L/h (median)		

Jeffery 1994 [33]	0.5 h	AN69 Filtral 10	n/r	200	CVVH (post)	1.5 L/h			CVVHD	1.5 L/h

Kellum 1998 [34]^g^	24 h	AN69 (0.6 m^2^)	UFH	150 to 200	CVVH (n/r)	2 L/h			CVVHD	2 L/h

Garcia-Fernandez 2000 [35]	24 h	High Flux polysulfone (0.6 m^2^) Bellco, Sorin Biomedica	UFH	100 to 150	CVVH (post)	0.71 L/h (median)	CVVHDF (post)	1 D + 0.56 F L/h (median)		

Maxvold 2000 [36]	24 h	Polysulfone hemofilt. HF-400, Renal Systems	UFH	4 (mL/kg/min)	CVVH (pre)	2^h ^L/h			CVVHD	2^h ^L/h

Wynkel 2004 [37]	24 h	AN69S, M100 (0.9 m^2^) changed daily	UFH or LMWH	150	CVVH (pre and post)^i^	1.5 L/h			CVVHD	1.5 L/h

Ricci 2006 [38]	Filter failure	AN69 (0.9 m^2^) multiflow 100, Hospel, UF coefficient with blood 25 mL/h/mm Hg × m^2^; 40 kDa cutoff	UFH	150 (F) and 135 (D) (medians)	CVVH (pre/post to keep filtration fraction <20%)	35 mL/kg/h (1.45 L/h pre + 1.5 L/h post (medians)); 19 (IQR 12.5, 28) h			CVVHD	35 mL/kg/h (2.15 L/h (median)); 37 (IQR 19.5, 72.5) h

Davies 2008 [39]	Filter failure	AN69 (Nephral 300ST, Hospal)	UFH	150 to 200	CVVH (pre)	35 mL/kg/h (mean 3.1 L/h); 8.6 ± 5.6 h	CVVHDF (pre)	1 D + 0.6 F L/h; 18.7 ± 3.1 h		

^a^Cessation refers to discontinuation of study renal replacement therapy by clinician discretion, or when a fixed time point was reached. For the crossover trials the fixed time point refers to the time prior and after the crossover (that is, duration of each mode). ^b^For filtration modes, "pre" and "post" refer to pre- and post-filter infusion of replacement fluid if reported. ^c^For trials of continuous renal replacement therapy, effluent flow rates are as reported in the publications assuming no net fluid removal and without adjustment for reduced clearance due to prefilter replacement fluid (for CAVH, CVVH, CAVHDF, or CVVHDF, or IDHF) except where noted [30]. ^d^For Morgera 2004 [24], patients were randomized to 1 of 4 groups: 1 or 2.5 L/h of CVVH or 1 or 2.5 L/h of CVVHD. ^e^For Maher 1988 [31], patients were also randomly crossed over to 2 additional CAVHD dose groups: 1.5 and 2 L/h of CAVHD. ^f^For Alarabi 1992 [32], patients were crossed over twice and received three treatments each over 24 hours, either CAVH-CAVHDF-CAVH or CAVHDF-CAVH-CAVHDF. We only included data from the first two treatments for each patient. ^g^For Kellum 1998 [34], we only included data from the 10/13 patients that received both treatments (3/13 patients died prior to being crossed over). ^h^For the pediatric patients in Maxvold 2000 [36], dose is expressed in L/h/1.73 m^2 ^body surface area. ^i^For Wynkel 2004 [37], patients were randomly assigned to receive three treatments each over 24 hours in one of the following orders: CVVHD/CVVHpre/CVVHpost, CVVHpre/CVVHpost/CVVHD, or CVVHpost/CVVHD/CVVHpre. ^j^For parallel group trials; and for crossover trials that specified duration of RRT based on filter failure and not a fixed duration of time. ^k^Calculated using mean weight of 59 kg obtained after author contact [27]. ^l^Data provided after author contact [28]. Randomized into three groups each treated for 8 hours per day: dialysis only ("sustained low efficiency daily dialysis" group in published abstract), diafiltration ("sustained low efficiency daily diafiltration" group), and filtration only ("predilution hemofiltration" group). F40s filter used for the dialysis-only group, and F80s for the diafiltration and filtration-only groups. Blood flow of 200 to 250 mL/min in the dialysis-only and diafiltration groups, and 250 to 300 mL/min in the filtration-only group. Filtration dose in the filtration-only group (in mL/min) was set at 1.25 × body weight (in kg) and body weight in this group was 64.4 ± 10.2 (mean ± standard deviation).

Abbreviations: CAVH, continuous arteriovenous hemofiltration; CAVHD, continuous arteriovenous hemodialysis; CAVHDF, continuous arteriovenous hemodiafiltration; CV, cardiovascular component of SOFA [65] score; CVVH, continuous venovenous hemofiltration; CVVHD, continuous venovenous hemodialysis; CVVHDF, continuous venovenous hemodiafiltration; d, days; equiv., equivalent; D, dialysis dose; F, filtration dose; h, hours; HCO_3_, serum bicarbonate concentration; IHD, intermittent hemodialysis; IHDF, intermittent hemodiafiltration; IQR, interquartile range; K, serum potassium concentration; kDa, kiloDalton; kg, body weight in kilograms; L, liter; LMWH, low molecular weight heparin; μM, micromolar; m^2^, meter squared; min, minute; mL, milliliter; mM, millimolar; mm Hg, millimeter of mercury; nM, nanomolar; n/r, not reported; post, post-filter addition of replacement fluid in hemofiltration modes; pre, pre-filter addition of replacement fluid in hemofiltration modes; RRT, renal replacement therapy; SOFA, mean sequential organ failure assessment score [65]; U/O, urine output; UFH, unfractionated heparin

All crossover trials were single-centered, used CRRT, and included few randomized patients (median 11.5 patients (range, 5 to 31)) [31-39]. Six of nine crossover trials [31,33,34,36-38] compared hemofiltration and hemodialysis at similar doses. Of these, only three trials reported post-filter addition of replacement fluid [33,37] or appropriately adjusted the rate of pre-filter replacement fluid to compare similar clearances [38]. Patients in one of these three trials [37] received three treatments (post-filter hemofiltration, pre-filter hemofiltration and hemodialysis) in random order. In another trial [31], patients received hemofiltration and three doses of hemodialysis in random order. For this trial, we excluded the two higher dose hemodialysis groups and retained the two matched-dose hemofiltration and hemodialysis groups.

In the remaining three crossover trials, hemofiltration was compared to a mixture of hemofiltration and hemodialysis at either higher [32,35] or lower [39] doses. In one of these trials [32], patients were crossed over twice and received their initial treatment a second time; only data from the first two treatments were included.

One parallel-group trial used different fixed doses based on weight ranges [26] while two others used weight-based dosing [27,30]. The parallel-group trials with sustained low efficiency RRT used fixed doses for the hemodialysis and hemodiafiltration groups [29] and weight-based dosing for the hemofiltration group [28]. One crossover trial used weight-based dose prescriptions for both interventions [38], while another used it only for hemofiltration [39]. The remaining trials used either fixed doses [22-25,31,33,34,36,37] or dose ranges not directly related to patient weight [21,32,35].

Among the parallel-group trials, RRT was discontinued at the clinicians' discretion [21-23,25,27,29], after a fixed duration [24,28], or when protocol-defined criteria were met [26,30]. The mean duration of RRT ranged from 2 to 8 days in four parallel-group CRRT trials [24,25,27,30] and just over 20 days in the parallel-group with intermittent therapy [23]. Among the crossover trials, patients were crossed over after a fixed time: 24 h for most trials [32,34-37] and 0.5 h for one trial [33]. Two trials crossed patients over after filter clotting or failure [38,39]. One trial did not report when patients were crossed over [31].

### Study quality (Table 3)

In the parallel-group trials that provided these data, all patients were analyzed according to the group to which they were initially assigned, and withdrawal of randomized patients from the mortality analysis either did not occur [26,27] or comprised ≤5% of randomized patients [21,23,30]. Caregiver blinding was not practical in any trial, given the nature of the intervention. Four trials reported [21,23,26,30] concealed allocation. In three trials reporting mortality [24,25,27] the authors informed us that they allocated patients to interventions in an alternating manner. The author of one trial informed us that the trial stopped early for benefit [26]. This trial was included only in the sensitivity analyses due to the use of differing doses and a mixture of hemofiltration and hemodialysis. Quality measures shown in Table 3 were generally not reported for the crossover trials, although in two crossover trials, authors reported that 3/13 (23%) [34] and 14/45 (31%) [39] patients were not crossed over to the other treatment. Carryover effects were generally ignored, which may have been reasonable given that the impact of clearance mode on solute removal should manifest relatively quickly [8], and subsequent clearance and concentration measurements were collected over a period of 12 to 24 hours after crossover in most trials. The trial with the shortest measurement period (30 minutes) provided a 10-minute equilibration period after crossover prior to data collection [33]. For one other trial [37], we included data collected over 12 hours but not starting until 12 hours after crossover. Most crossover trials reported using paired analyses [33-37,39], but none provided individual patient data or mean within-patient difference data. Therefore, we were restricted to using group-specific means to perform meta-analyses, as discussed in the Methods.

**Table 3:** Risk of bias of included trials

Trial	Sequence generation	Concealment of allocation	Trial stopped early for benefit	Intention to treat analysis	Post-randomization withdrawals for mortality analysis (parallel group trials) or not crossed over
					

***Parallel group***					

Davenport 1993 [21]	List of random numbers^a^	Yes^a^	No	Yes	Yes - 1/12 CVVHD(lost to follow up)

Alamartine 1994 [22]^a^	n/r	n/r	n/r	n/r	n/r

Pettila 2001 [23]	Computer generated	Yes^a ^(closed envelopes)	No	Yes	Yes - 1/18 IHD(consent withdrawn)

Morgera 2004 [24]	Alternating patients^a^	No	No	Yes	n/r

Daud 2006 [25]	Alternating patients^a^	No	No	Yes	n/r

Saudan 2006 [26]	Computer generated	Yes (sequentially numbered sealed opaque envelopes)	Yes^a^	Yes	No

Chang 2009 [27]	Alternating patients^a^	No	No^a^	Yes	No^a^

Ratanarat 2009 [28]	List of random numbers^a^	n/r	n/r	n/r	n/r

Ratanarat 2012 [29]	List of random numbers	n/r	n/r	n/r	n/r

OMAKI 2012 [30]	Computer generated	Yes (sequentially numbered sealed opaque envelopes)	No	Yes	Yes - 1/39 CVVHD(inclusion mistake)

***Crossover***					

Maher 1988 [31]	n/r	n/r	n/r	n/r	n/r

Alarabi 1992 [32]	n/r	n/r	n/r	n/r	n/r

Jeffery 1994 [33]	n/r	n/r	n/r	n/r	n/r

Kellum 1998 [34]	n/r	n/r	n/r	n/r	Yes - 2 CVVH and 1 CVVHD/13 died prior to crossover

Garcia-Fernandez 2000 [35]	n/r	n/r	No	n/r	n/r

Maxvold 2000 [36]	n/r	n/r	n/r	n/r	n/r

Wynkel 2004 [37]	n/r	n/r	n/r	n/r	n/r

Ricci 2006 [38]	n/r	n/r	n/r	n/r	n/r

Davies 2008 [39]	n/r	n/r	n/r	n/r	Yes - 14/45 not crossed over

^a^Obtained after author contact. Abbreviations: CVVH, continuous venovenous hemofiltration; CVVHD, continuous venovenous hemodialysis; IHD, intermittent hemodialysis; n/r, not reported.

### Clinical outcomes

**Figure 2:**
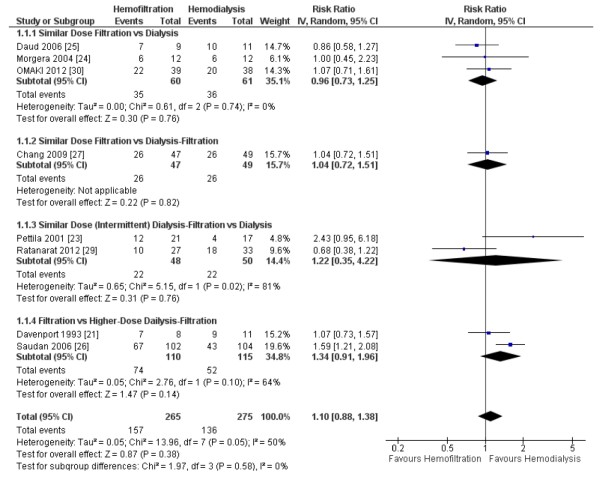
**Effect of hemofiltration vs. hemodialysis RRT on mortality**. The pooled risk ratio was calculated using a random-effects model. Weight refers to the contribution of each study to the overall estimate of treatment effect. Abbreviations: CI, confidence interval; IV, inverse variance.

The three parallel group trials that compared similar doses of hemofiltration to hemodialysis were included in the primary analysis and reported either ICU [24,25] or hospital [30] mortality. Five additional parallel-group trials comparing similar [23,27,29] or different [21,26] doses of hemodiafiltration to either hemodialysis or hemofiltration reported mortality data and were included in the sensitivity analysis. They reported hospital [21,23], 28-day [27,29], or 90-day [26] mortality. We did not identify differences in pooled mortality in the primary (RR 0.96, 95% CI 0.73 to 1.25, *P *= 0.76; three trials, 121 patients) or sensitivity (RR 1.10, 95% CI 0.88 to 1.38, *P *= 0.38; eight trials, 540 patients) analyses (Figure 2). Statistical heterogeneity was absent (*I^2 ^*= 0%) and moderate (*I^2 ^*= 50%), respectively. Visual inspection of the funnel plot for the sensitivity analysis did not suggest publication bias.

Dialysis dependence in survivors was not different between groups (primary analysis: RR 1.33, 95% CI 0.35 to 5.08, *P *= 0.67; two trials [25,30], 37 surviving patients; sensitivity analysis: RR 0.95, 95% CI 0.44 to 2.04, *P *= 0.89; four trials [25-27,30], 177 surviving patients), with no statistical heterogeneity (*I^2 ^*= 0%). Two trials reported no differences in SOFA scores 72 hours after starting therapy (primary analysis: RoM 1.02, 95% CI 0.89 to 1.16, *P *= 0.79; one trial, 63 patients [30]; sensitivity analysis: RoM 1.00, 95% CI 0.61 to 1.64, *P *= 0.99; two trials, 124 patients [27,30]). One trial reported lower SOFA scores in the hemofiltration group primarily between four and seven days, driven primarily by the cardiovascular component (reflecting lower vasopressor requirements); however, this decrease was not statistically significant [30]. Another trial reported no difference in norepinephrine doses or changes in the multi-organ dysfunction score (MODS) [66] or APACHE II score between continuous hemofiltration and hemodialysis groups over the first 72 hours [24]. One parallel-group trial, comparing intermittent hemodiafiltration to hemodialysis, reported similar improvements in MODS between groups over 10 days [23], and another comparing sustained low efficiency dialysis to diafiltration reported similar improvements in blood pressure between groups over three days [29].

**Figure 3:**
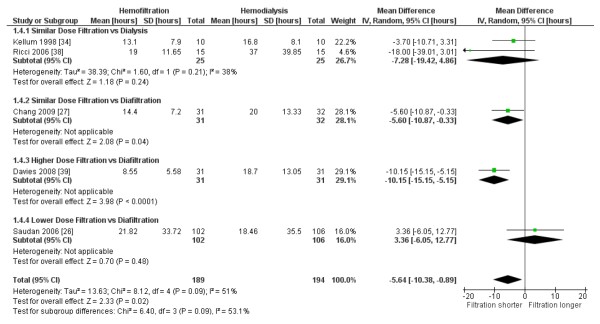
**Effect of hemofiltration vs. hemodialysis on filter life**. The pooled mean difference was calculated using a random-effects model. Weight refers to the contribution of each study to the overall estimate of treatment effect.

Pooled data from two small crossover trials using similar dose CVVH vs. CVVHD [34,38] suggest that hemofiltration may shorten the time to filter failure, although only the RoM result achieved statistical significance (MD -7.3 hours, 95% CI -19.4 to +4.9, *P *= 0.24, *I^2 ^*= 38%; RoM 0.67, 95% CI 0.45 to 0.99, *P *= 0.04, *I^2 ^*= 7%; n = 50). Incorporating the results of one parallel-group trial using similar dose CVVH vs. hemodiafiltration (CVVHDF) [27] produced a pooled result that was significant for both effect measures (MD -5.4 hours, 95% CI -9.6 to -1.3 hours, *P *= 0.01; RoM 0.70, 95% CI 0.56 to 0.88, *P *= 0.003; *I^2 ^*= 0% in both analyses; n = 113). Recognizing that higher dose may also affect filter life, two other trials comparing non-equivalent doses in the two treatment arms demonstrated shorter time to filter clotting in the higher dose group. One crossover trial comparing higher dose CVVH to CVVHDF [39] demonstrated an even greater decrease in filter life in the CVVH group, and one parallel group trial comparing lower dose CVVH to CVVHDF [26] demonstrated a non-statistically significant shorter time to filter clotting in the higher-dose group (Figure 3). Including the data from trials with non-equivalent doses in the two treatment arms in the pooled analysis resulted in a similar shortened time to filter failure in the hemofiltration group (MD -5.6 hours, 95% CI -10.4 to -0.9 hours, *P *= 0.02; RoM 0.69, 95% CI 0.50 to 0.95, *P *= 0.02; five trials, 383 patients), with higher heterogeneity (*I^2 ^*= 51 to 66%). All these trials used unfractionated heparin anti-coagulation and pre-filter replacement fluid except for one trial that used a mixture of pre- and post-filter replacement to keep the filtration fraction <20% [38]. This reduction in filter survival time of about one-third is equivalent to a 50% increase in filters required for hemofiltration compared to hemodialysis.

### Clearances of small and large molecules (Table 4)

Few molecules were examined in more than one study, and analyses included few patients. In general, small molecule clearance (for example, urea, phosphate and creatinine) was similar between hemofiltration and hemodialysis, whereas hemofiltration achieved higher clearance of larger molecules (up to around 20 kiloDaltons (kDa)). Pooled data from two studies [24,34] showed similar clearance of interleukin (IL)-6 between modes, but statistical heterogeneity was high. Single studies also found that hemofiltration delivered significantly higher clearances of protein and albumin (using a high (60 kDa) cut-off filter) [24], and almost all amino acids in one study examining pediatric patients receiving amino acid supplementation [36].

**Table 4:** Clearance measurements of hemofiltration vs.

		Change in clearance hemofiltration vs hemodialysis^a^			
		
Molecular substance	Number of trials; number of patients randomized	Effect estimate	95% confidence interval	*P*-value	Heterogeneity (*I^2^*)
**Smaller molecules**					

Urea (60 Da)	4 [33,36-38]; 49	+1%^b^	-2% to +3%	0.60	0%

Phosphate (95 Da)	1 [37]; 18	0%^c^	-4% to +4%	1.00	n/a

Creatinine (113 Da)	3 [33,37,38]; 43	+1.8%^b^	-0.4% to +4.1%	0.12	0%

Uric acid (168 Da)	2 [33,37]; 28	+4%	+1% to +7%	0.01	0%

**Larger molecules**					

Vancomycin (1.8 kDa)	1 [33]; 10	+18%	+8% to +28%	0.0003	n/a

β_2_-microglobulin (11.8 kDa)	2 [37,38]; 33	+94%^d^	+78% to +112%	<0.0001	0%

IL-1 Receptor Agonist (16-18 kDa)	1 [24]; 12	+77%^e,f^	+24% to +153%	0.002	n/a

Retinol Binding Protein (21.2 kDa)	1 [37]; 18	+42%	+4% to +94%	0.03	n/a

IL-6 (26 kDa)	2 [24,34]; 22	+6%^f,g^	-62% to +191%	0.91	89%

hemodialysis

^a^Using RoM. ^b^Results are unchanged if two trials either using different ultrafiltration rates [31] or comparing different doses of hemofiltration vs a mixture of hemodialysis and hemofiltration [21] are also included after accounting for the different flow rates (urea clearance +1% (95% CI -2% to +3%, *P *= 0.58, *I^2 ^*= 0%, six studies [21,31,33,36-38], 74 patients) and creatinine clearance +1.9% [95% CI -0.3% to +4.1%, *P *= 0.09, *I^2 ^*= 0%, five studies [21,31,33,37,38], 68 patients)). ^c^Results unchanged if data from the filtration-only vs dialysis-only groups in the sustained low efficiency RRT trial [28] are added: +31% (95% CI -35% to +165%, *P *= 0.45, *I^2 ^*= 72%, two studies [28,37], 30 patients). ^d^One of these studies [38] also measured adsorptive clearance of β_2_-microglobulin and found negligible (<1%) clearance occurred via adsorption to the filter and this was similar between hemofiltration and hemodialysis. ^e^Comparing higher-dose (2.5 L/h) groups only. Comparing lower-dose (1 L/h) groups in the same study [24] showed a smaller but still statistically significant increased clearance of IL-1 Receptor Agonist +28% (95% CI +10% to +50%%, *P *= 0.002). ^f^The medians and ranges reported in this trial [24] were converted to means and standard deviations using published guidelines [81]. ^g^Including the higher-dose (2.5 L/h) groups for Morgera 2004 [24] only. Including also the lower-dose (1 L/h) groups for this trial [24] produced similar results -2% (95% CI -46% to +80%%, *P *= 0.96, *I^2 ^*= 0%, two studies [24,34], 34 patients]. Abbreviations: CI, confidence interval; Da, Daltons; *I^2^*, *I^2 ^*heterogeneity measure; IL, interleukin; kDa, kiloDaltons; n/a, not applicable; RoM, ratio of means [17]; RRT, renal replacement therapy.

The impact of clearance mode on serum concentrations of various solutes of interest was reported even less frequently. One crossover trial [34] found the concentration of tumor necrosis factor (TNF) α (but not IL-6, IL-10, SL-selectin, and endotoxin) to be significantly lower in the patients during hemofiltration. A second crossover trial [35] found no differences in concentrations of mediators of endothelial activation. One parallel-group trial [25] measured a larger decrease in IL-6 and smaller increase in TNFα concentration in the hemofiltration group, but these cytokines were only measured in one patient treated with hemofiltration and two patients treated with hemodialysis. Finally, the crossover trial of pediatric patients receiving amino acid supplementation [36] reported lower serum concentrations of amino acids in association with higher clearances in the hemofiltration group.

## Discussion

This systematic review and meta-analysis highlights the paucity of data from randomized controlled trials comparing hemofiltration to hemodialysis in the treatment of AKI. Considering clinical outcomes of hemofiltration in parallel-group RCTs, there was no indication of improved mortality or organ dysfunction, although confidence intervals were wide. Our meta-analysis suggests that hemofiltration shortens filter life by about five to six hours (or one-third of total mean filter time). Based primarily on crossover RCTs, we found that hemofiltration increases the clearance of medium to larger molecules compared to hemodialysis. Almost no studies determined whether the enhanced middle-molecule clearance attributed to hemofiltration actually led to lower serum concentrations.

The trials reporting on filter failure rates used primarily pre-filter addition of replacement fluid. Comparing pre-filter to post-filter addition, one trial [37] included in this meta-analysis and other studies [67-69] have demonstrated that replacement fluid requirements are about 15 to 20% higher to achieve similar small molecule clearance rates at doses of around 20 mL/kg/h. Our finding of a 33% shorter time to filter failure with hemofiltration, combined with its higher fluid requirements to achieve similar small molecule clearance, implies that hemofiltration may consume more resources than hemodialysis. This hypothesis merits further evaluation in a formal cost analysis.

**Figure 4:**
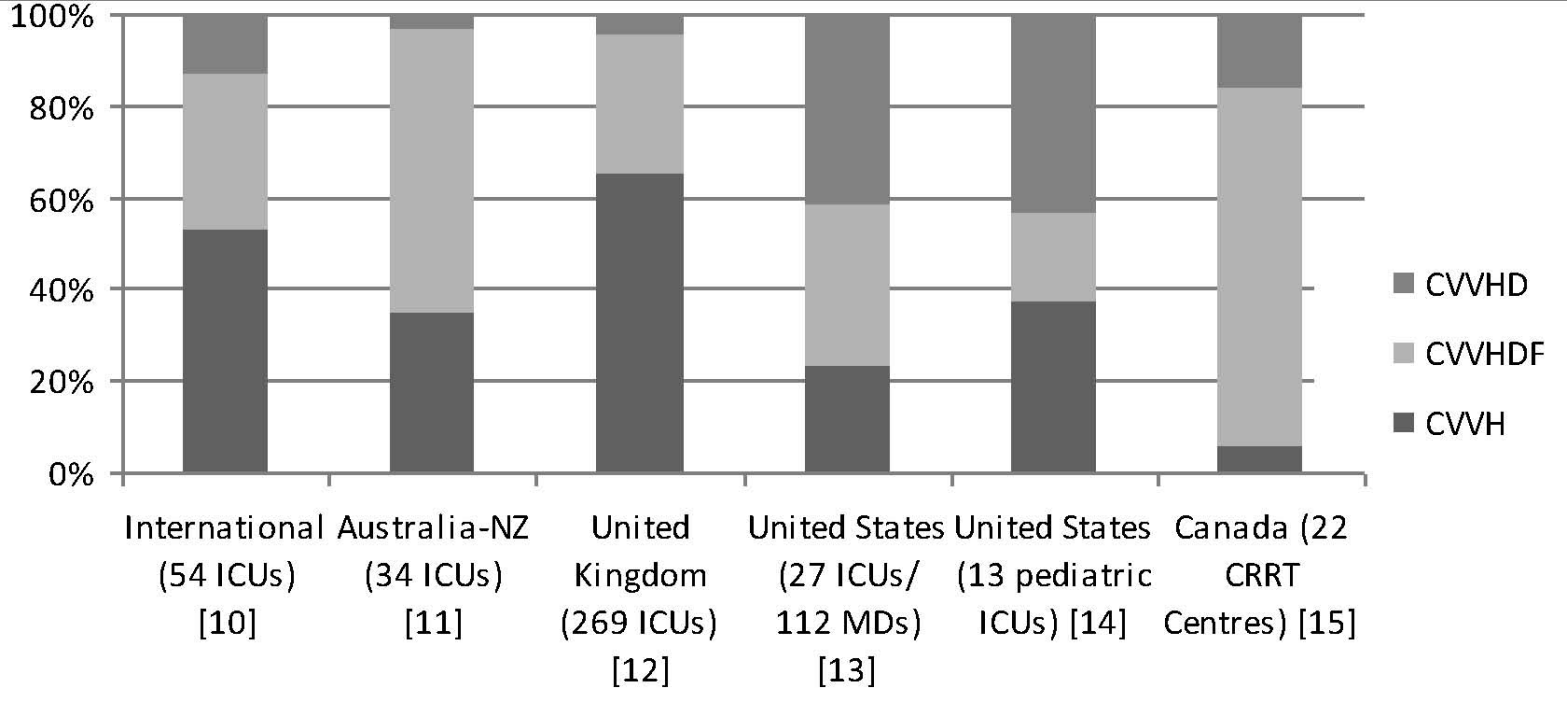
**Distribution of mode of RRT used in different countries/regions based on practice surveys**. Abbreviations: CRRT, continuous renal replacement therapy; CVVH, continuous venovenous hemofiltration; CVVHD, continuous venovenous hemodialysis; CVVHDF, continuous venovenous hemodiafiltration; ICU, intensive care unit; MD, medical doctor; NZ, New Zealand; RRT, renal replacement therapy; UK, United Kingdom; US, United States.

Comparing the published practice survey data in different countries and regions [10-15] (summarized in Figure 4), it appears that physicians tend to use a mode with at least some hemofiltration (that is, either CVVH or CVVHDF), perhaps anticipating additional benefit associated with hemofiltration. However, the small number of trials, randomized patients and events does not support this belief. A well-designed and adequately powered trial would be necessary to establish the superiority of hemofiltration.

Strengths of our review include methods to minimize bias, such as a comprehensive literature search, duplicate data abstraction, consideration of important clinical outcomes, and inclusion of additional methodological or clinical information from authors. The primary limitation is the small number and size of RCTs comparing pure hemofiltration to pure hemodialysis at similar doses. Sensitivity analyses, including trials whose arms also varied with respect to dose, gave similar results, as expected given recent large trials [4,5] and meta-analyses [6,7] that found similar outcomes with different RRT doses. In addition, trials varied in the modality of RRT used, timing of initiation, and types of filters and blood flows, although recent meta-analyses have not found differential outcomes based on these factors [70-73]. We did not consider comparisons of blood clearance modes with peritoneal dialysis [74,75], which is used in some areas of the world to treat acute kidney failure. Finally, heterogeneity may have been underestimated because these tests are underpowered when there are few trials. Although hemofiltration is of particular interest in patients with sepsis, in whom pro-inflammatory mediators are increased, there was insufficient data to conduct a subgroup analysis in these patients. In addition, we did not examine the role of hemofiltration vs hemodialysis in patients with sepsis who have not yet developed AKI [40] or the role of hemofiltration compared to no RRT [76-79].

## Conclusions

Pooled data from a few randomized trials suggest that hemofiltration increases the clearance of medium to larger molecules without improving clinical outcomes, though confidence intervals are wide. Hemofiltration may also reduce filter life. This latter finding, together with the increased replacement fluid requirements to achieve equivalent small-molecule clearance when pre-filter replacement is used, suggests that hemofiltration may be more expensive than hemodialysis. Our findings support the need for additional pilot data [80] to evaluate the impact of hemofiltration vs. hemodialysis on intermediate outcomes, such as vasopressor requirements, that may serve as valid surrogates for important clinical outcomes that could subsequently be evaluated in a large definitive trial.

## Key messages

• Few randomized controlled trials have compared hemofiltration vs hemodialysis for the treatment of acute kidney injury.

• Pooling the results from these trials does not suggest beneficial clinical outcomes of hemofiltration vs hemodialysis, but confidence intervals are wide.

• Compared to hemodialysis, hemofiltration may increase clearance of medium to larger molecules, but may also shorten the time to filter failure.

• Additional pilot trials are needed to evaluate the impact of hemofiltration vs. hemodialysis on intermediate outcomes, such as vasopressor requirements, that may serve as valid surrogates for important clinical outcomes that could subsequently be evaluated in a large definitive trial.

## Abbreviations

AKI: acute kidney injury; APACHE: acute physiology and chronic health evaluation; CI: confidence interval; CRRT: continuous renal replacement therapy; CVVH: continuous veno-venous hemofiltration; CVVHD: continuous veno-venous hemodialysis; CVVHDF,continuous veno-venoous hemodiafiltraion; HD: hemodialysis; HF: hemofiltration; *I^2^*: *I^2 ^*heterogeneity measure; ICU: intensive care unit; IL: interleukin; kDa: kiloDalton; MD: mean difference; MODS: multi-organ dysfunction score; RCT: randomized controlled trial; RoM: ratio of means; RR: relative risk; RRT: renal replacement therapy; SAPS: simplified acute physiology score; SOFA: sequential organ functional assessment; TNF: tumor necrosis factor

## Competing interests

The authors declare that they have no competing interests.

## Authors' contributions

JF and NA acquired and analyzed the data. JF wrote the first draft of the manuscript. All authors were involved with the conception and design of the study, interpretation of the data, and critical revision of the manuscript for important intellectual content. All authors have read and approved the manuscript for publication.
